# Intercostobrachial Nerves as a Novel Anatomic Landmark for Dividing the Axillary Space in Lymph Node Dissection

**DOI:** 10.1155/2013/279013

**Published:** 2013-01-20

**Authors:** Jianyi Li, Yang Zhang, Wenhai Zhang, Shi Jia, Xi Gu, Yan Ma, Dan Li

**Affiliations:** ^1^Department of Breast Surgery, Shengjing Hospital of China Medical University, Shenyang, Liaoning 110004, China; ^2^Ultrasound Diagnosis Center, Shengjing Hospital of China Medical University, Shenyang, Liaoning 110004, China; ^3^Pathological Diagnosis Center, Shengjing Hospital of China Medical University, Shenyang, Liaoning 110004, China

## Abstract

*Purpose*. Our aim was to assess the feasibility of using the intercostobrachial nerves (ICBNs) as a possible new anatomic landmark for axillaries lymph node dissection in breast cancer patients. *Background Data Summary*. The preservation of ICBN is now an accepted procedure in this type of dissection; however, it could be improved further to reduce the number of postoperative complications. The axillary space is divided into lower and upper parts by the ICBN—a thorough investigation of the metastasis patterns in lymph nodes found in this area could supply new information leading to such improvements. *Methods*. Seventy-two breast cancer patients, all about to undergo lymph node dissection and with sentinel lymph nodes identified, were included in this trial. The lymph nodes were collected in two groups, from lower and upper axillary spaces, relative to the intercostobrachial nerves. The first group was further subdivided into sentinel (SLN) and nonsentinel (non-SLN) nodes. All lymph nodes were tested to detect macro- and micrometastasis. *Results*. All the sentinel lymph nodes were found under the intercostobrachial nerves; more than 10 lymph nodes were located in that space. Moreover, when lymph nodes macrometastasize or micrometastasize above the intercostobrachial nerves, we also observe metastasis-positive nodes under the nerves; when the lower group nodes show no metastasis, the upper group is also metastasis free. *Conclusions*. Our results show that the intercostobrachial nerves are good candidates for a new anatomic landmark to be used in lymph node dissection procedure.

## 1. Introduction

The axillary lymph node (ALN) status represents one of the most important prognostic factors in breast cancer patients and determines, among other parameters, the type of subsequent adjuvant treatment [1, 2]. Lymph nodes in axillary space are traditionally divided into 3 groups by pectoralis minor (level I, level II, and level III), according to the rule of lymph nodes metastasis [3]. It is the accepted basic principle for axillary lymph node dissection (ALND) of breast cancer that lymph nodes should be extracted from level I to level III, step by step [4]. The postoperative risk of arm lymphedema increases with the increasing axillary space level during the dissection [5]. On the other hand, the necessity of intercostobrachial nerves (ICBNs) preservation is now accepted by the surgeons and has become the standard procedure in such dissections, reducing the postoperative skin numbness and loss of feeling in the upper arm [6]. The ICBN is nearly parallel to the axillary vein, at the distance of about 1.5 cm [7]. It naturally divides the axillary space into the lower and upper parts. The upper part includes level III, a large part of level II, and a small part of level I; if the tissue between the ICBN and axillary vein is preserved, the postoperative lymphedema of arm should be prevented. Therefore, it would be useful to decide whether ICBN could serve as the anatomic landmark for dividing the axillary lymph nodes into groups, with a practical application in mind. In the current study, the data of 72 patients with breast cancer are analyzed to identify the feasibility of such assignment and potential clinical significance of ICBN in dividing the axillary space. The relationship between lymph node metastasis and ICBN, the number of nodes under the ICBN, and the relationship between sentinel nodes and ICBN is also examined.

## 2. Patients and Methods

### 2.1. Patients

Between July 2009 and May 2010, a total of 86 patients with breast cancer, about to undergo the axillary lymph node dissection in Shengjing Hospital (SJHCMU), were enrolled in this study. Inclusion criteria for the study were (1) infiltrating duct carcinoma (IDC) identified by a pathologist, (2) no prior history of breast cancer or other malignancies, (3) no neoadjuvant therapy, (4) no pregnancy, (5) sentinel lymph nodes (SLNs) identified using methylene blue dye (MBD). Fourteen patients did not meet the criteria and were therefore excluded; seventy-two patients met the inclusions criteria. All the patients agreed to the axillary lymph node dissection (ALND) and signed the Informed Consent Sheet, even if the SLN was negative (as judged by using the frozen section method intraoperatively). All the lymph nodes dissections were performed by the same surgical team of China Medical University affiliated Shengjing Hospital (SJHCMU). The study was approved by the Local Ethics Committee.

### 2.2. Lymph Nodes Groups and Operative Techniques

**Figure 1:**
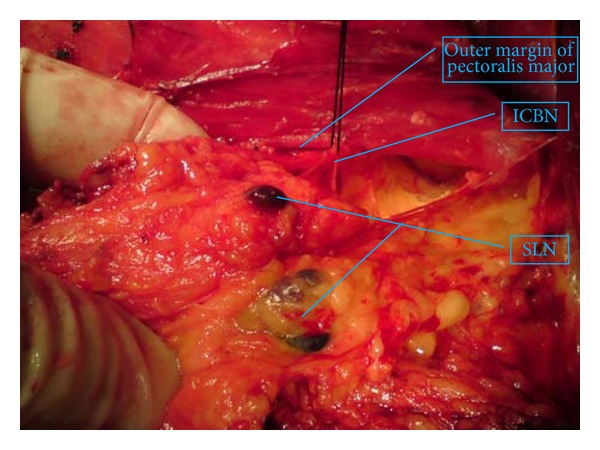
It is considered SLN; those blue LNs are stained by MBD, and there are two SLNs under the ICBN and besides the outer margin of pectoralis minor. Those LNs belong to lower LNs and level I.

**Figure 2:**
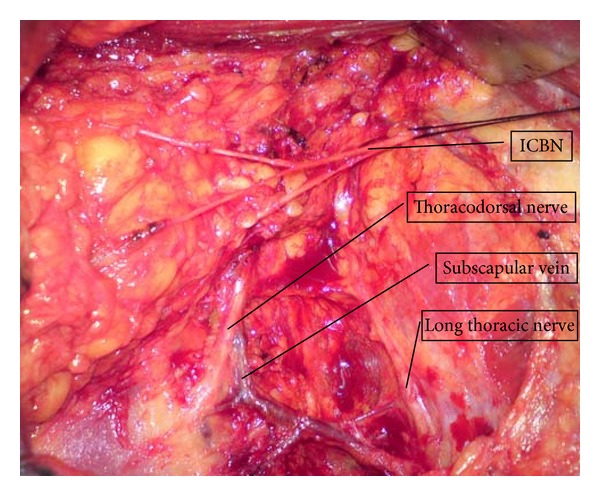
The extent of dissection under the ICBN. The ICBN was revealed completely in axillary space, and the LNs adjoined with ICBN belonged to the lower LNs. It was necessary to reveal and reserve the long thoracic and thoracodorsal nerves in the operation.

Sentinel lymph node mapping was performed using methylene blue dye (MBD), as described previously [8]. Briefly, 3-4 mL MBD (1.25 mg/mL) was injected into tumor bed and areola of breast 15 minutes before the dissection. If the tumor was located in the outer quadrant, two-thirds of the dose of MBD was injected into tumor bed, and one-third into areola of breast; the dosage was distributed the other way round for tumors located in the inner quadrant. All the patients underwent ALND according to the principle of level I to level III dissection, but the lymph nodes were divided into three groups, as described earlier. All the blue staining nodes were identified as sentinel nodes (SLNs, Figure 1). Nodes defined as non-SLNs were located under the intercostobrachial nerves and did not show blue staining. All the nodes above the ICBN were assigned to the upper lymph node group. This last group included a small part of level I, a large part of level II, and level III. Because there were no blue staining nodes above the intercostobrachial nerves, the SLN and non-SLN constituted the lower lymph node group. The clearance of lower nodes' group has to be defined to preserve the ICBN, a large part of long thoracic and thoracodorsal nerves under the ICBN (Figure 2).

### 2.3. Examination of Lymph Nodes Pathology

The frozen sections of SLN were routinely used intraoperatively to determine which level should be dissected. The patients with SLN macrometastases in frozen sections underwent immediately level II or level III dissection; others, without SLN macrometastasis, were submitted to level I or level II axillary lymph node dissection. The extracted lymphatic tissue was formalin fixed and embedded in paraffin for histological analysis. The tissue was then examined using step sectioning at a cutting interval of 250 *μ*m. Step sections were stained with hematoxylin & eosin (H&E). If no carcinoma cells were detected in the nodes, immunohistochemistry with cytokeratin antibody CK-22 (Santa Cruz, CA, USA), using a standard immunoperoxidase method (ABC Elite kit), was performed [9]. Micrometastasis was defined as tumor of the size exceeding 0.2 mm and less than or equal to 2 mm in diameter, according to the American Joint Committee of Cancer (AJCC) classification. Hence, isolated tumor cells or tumor cell clusters measuring less than or equal to 0.2 mm in diameter did not meet the definition of micrometastases. Therefore, the patients with such clusters were considered as micrometastasis negative. All the previous analysis was performed by a pathologist from the Breast Group of Pathology Diagnosis Center of SJHCMU.

### 2.4. Statistical Analysis

The data are represented by the mean values or the frequency tables, depending on the data type. Statistical analysis was performed using SPSS 17.0 (SPSS, Chicago, IL, USA).

## 3. Results

**Figure 3:**
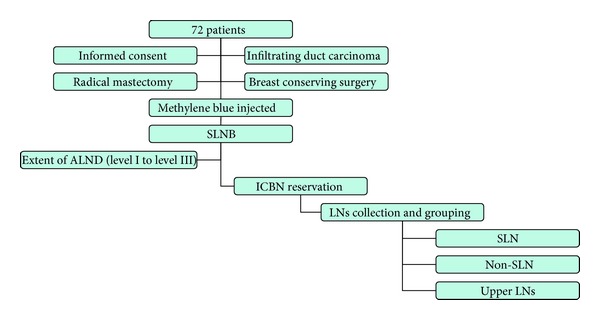
72 patients have accomplished follow chart.

From July 2009 to May 2010, the sentinel node biopsies were performed on 86 breast cancer patients. The overall SLN identification rate was 95.3% (82/86). Fourteen patients did not meet the inclusion criteria for this study because four patients have not been SLN stained by MBD, and ten patients' tumors were not infiltrating duct carcinoma, as judged by the postoperative pathological diagnosis (Figure 3). The characteristics of the 72 patients are listed in Table 1. The median age was 49 years (range 33–72). The median tumor size was 1.9 mm (range 0.9–5.5). About half of the tumors were located in the outer-upper quadrant of breast. The preoperative axillary assessment was performed using 3 methods: palpation, Doppler ultrasound, and molybdenum target photography (MTP). If the lymph node tumescence was identified by any one of these methods, the axillary nodes were considered to be positive preoperatively; about 40% of patients were found to have metastatic lymph nodes.

The blue staining nodes were not detected above ICBN in any of the patients. The nodes below the nerves were divided into SLN and non-SLN groups; the lymph node numbers in each group are shown in Table 2. The total number of axillary space lymph nodes examined was 1503: 232 SLNs, 685 non-SLNs, and 586 upper group lymph nodes. The average number of axillary lymph nodes per patient was 20.94; 3.22 in SLN group, 9.51 in non-SLN group, and 8.14 in the upper group. The mean for lower group nodes' number was 12.74, which exceeds the required number of 10 and is, therefore, sufficient for assessing the axillary node status.

All the lymph nodes collected were formalin fixed and embedded in paraffin for histological analysis. The number of patients with macrometastasis-positive (LN+) and macrometastasis-negative (LN−) nodes in different groups is shown in Table 3. There were 19 patients with one macrometastasis node in non-SLN subgroup, 15 showing metastasis in SLN subgroup, and 3 in upper LN group.  Table 4 contains the data for the individual patients with macrometastasis, identified using H&E. Metastasis in non-SLN and upper group nodes was found in one case (^#^), in three cases we see metastasis in non-SLN group only (*), and two patients show metastasis positive nodes in all the groups (^§^). In the remaining 13 metastasis cases, the positive nodes were found in the lower space groups. To summarize, in patients with metastasis-negative lower nodes, the upper group nodes were also metastasis free; in cases with the upper metastasized nodes, the lower nodes showed positive staining as well. Within the lower space node group, in cases with the positive-staining SLN, the non-SLN subgroup was also positive.

All the macrometastasis-negative nodes were stained with CK-22 to detect micrometastasis; the results are shown in Table 5. There were 24 patients with micrometastasis nodes in non-SLN group, 12 patients showed metastasis in SLN group, and 3 in the upper group.

Table 6 shows the results for individual patients where micrometastasis positive nodes were identified by CK-22 staining in at least one of the node groups. We found micrometastasis in nodes from all three groups in one patient only (^#^) and micrometastasis limited to sentinel nodes also in one patient (^¥^). In two cases, we found positive nodes in both non-SLN group and upper node group (*). In 10 cases, there were positive-staining nodes in SLN and non-SLN groups (^§^); only non-SLN group positives were discovered in other 10 patients (^$^).

In most cases, the upper group nodes do not show micrometastasis. In cases where it does occur, metastasis-positive nodes are also found in one of the lower space node groups.

## 4. Discussion

**Figure 4:**
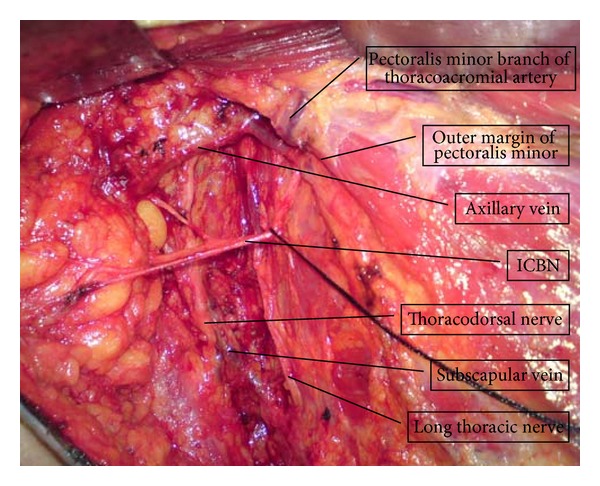
The PMBTA is located at the outer margin of pectoralis minor, and it seems to be vertical to ICBN and axillary vein. The vertical dimension from axillary vein to PMBTA is about 1.5 cm.

According to Lloyd's study of skip metastases in lymph nodes (1989), the problems associated with axillary lymph node dissection in breast cancer should be all but solved [3]. It has been long accepted by most surgeons that ALND should precede from level I to level III, step by step, according to the results of SLN biopsy, and that 10 lymph nodes should be obtained from the axillary space [10, 11]. However, the arm lymphedema still remains an important and not uncommon postoperative complication, and there are no effective measures to deal with this problem [12]. A meta-analysis suggested that mastectomy, extent of axillary dissection, radiation therapy, and presence of positive nodes can all increase the risk of developing arm lymphedema after breast cancer treatment [13]. Some existing studies show that the increased incidence of lymphedema is associated with the extent and degree of ALND, more than with any other factor [14]. McLaughlin's study suggests that the lymphedema incidence can be reduced from 16% to 5% by using sentinel node biopsy, while more than 16% of patients after ALND (level I to level III) suffer from arm lymphedema [15]. Therefore, the question of what constitutes an adequate axillary dissection in breast cancer treatment still remains largely unanswered. On the other hand, the procedure of ICBN preservation provides a new potential anatomic landmark in axillary lymph node dissection. It has been reported that in 86% of cases, ICBN is found in the second intercostals space in axillary [16]. In the current study, we found intercostobrachial nerves easily in that location, in all cases. Should ICBN's location vary, the pectoralis minor branch of thoracoacromial artery (PMBTA) can substitute ICBN as anatomic landmark (Figure 4). The technique of ICBN preservation is accepted by the majority of surgeons and easily followed; so, from the practical point of view, there should be no problems in utilizing those nerves as a new landmark for dividing the axillary space. The results displayed in Table 2 show that sentinel lymph nodes are always located under the ICBN, and the number (12.74) of lymph nodes under ICBN exceeds 10. Some reports suggest that the sentinel nodes are always located near the lateral thoracic artery [17]. In our study, the number of non-SLNs, also in the lower node group, is 9.51, which is close to 10. In general, it can be assumed that if there are some SLNs located under ICBN, the number of lower group nodes will exceed 10. This means that enough lymph nodes can be obtained under the ICBN to assess axillary node status. Goldberg considers that there is no correlation between number of lymph nodes removed and change in the upper extremity circumference or incidence of lymphedema. His data suggest that other factors, such as the global disruption of the lymphatic channels during axillary lymph node dissection, play a larger role in development of lymphedema than does the number of nodes removed [18].

In summary, taking into account our results, we could formulate two interesting rules for lymph node metastasis in lower and upper groups in axillary space divided by ICBN. If the lower group nodes are negative by H&E and CK-22 staining, the upper nodes are also negative (Tables 3 and 5); if the upper group nodes are positive by H&E and CK-22 staining, the lower nodes are positive too (Tables 4 and 6). Although skip metastasis had been identified in some previous studies, we did not detect any evidence of skip metastasis in the current analysis, possibly because of a small number of cases [19]. The results suggest that the lymph node metastases progress from lower to upper and from outer to inner areas, stepwise, and the probability of skip metastasis might be quite low. This rule for lymph node metastases can be probably applied only to infiltrating duct carcinoma; other cancers or sarcomas should be investigated separately. Most surgeons agree that the destruction of lymph vessel draining in the arm during ALND procedure is the main reason of postoperative lymphedema [20]. Existing studies suggest that the lymph draining in the arm is closely associated with axillary vein [21]. If the lymphatic and fatty tissues between ICBN and axillary vein could be preserved, the postoperative arm lymphedema would be prevented in most cases. Therefore, it is of a potential clinical significance that the axillary space can be considered, for practical purposes, subdivided by ICBN. Long-term follow-up studies should show that preservation of tissue above the ICBN can substantially reduce or even completely prevent the postoperational arm lymphedema. If the outer margin of pectoralis minor is replaced by ICBN as the anatomic landmark between level I and level II, the new level I in ALND procedure for early stage breast cancer can be defined with sufficient number of lymph nodes to assess the axillary lymph node status. At the same time, the lymph vessel draining in the arm will not be disturbed, avoiding the postoperational lymphedema complications. This, of course, can be only confirmed by thorough, long-term follow-up studies.

## Figures and Tables

**Table 1:** Characteristics of patients with breast cancer (*n* = 72).

Parameters	Number	Percent
Age (years)		
Median	49	
Range	(33–72)	
Tumor size (cm)		
Median	1.9	
Range	(0.9–5.5)	
Quadrant		
Areolar	2	2.8
Outer upper	37	51.4
Outer lower	15	20.8
Inner lower	7	9.7
Inner upper	11	15.3
Operation		
Mastectomy	61	84.7
Tumorectomy	11	15.3
Axillary assessment		
Positive	29	40.3
Negative	43	59.7
Histological grading		
G1	17	23.6
G2	45	62.5
G3	10	13.9
Estrogen receptor status		
Positive	37	51.4
Negative	35	48.6
Progesteron receptor status		
Positive	31	43.1
Negative	41	56.9
Her2 receptor status		
Positive	28	38.9
Negative	44	61.1

**Table 2:** Lymph node numbers in different node groups.

	Lower LN			Upper LN group	Total
	SLN	Non-SLN	SLN + non-SLN
Sum of LN	232	685	917	586	1503
Mean number of nodes per patient	3.22 ± 1.47	9.51 ± 3.34	12.74 ± 3.50	8.14 ± 3.20	20.94 ± 4.83

SLN: sentinel lymph nodes group; non-SLN: nonsentinel lymph nodes group.

**Table 3:** Macro-metastasis as demonstrated using H&E. Number of patients with macro-metastasis-positive (LN+) and macro-metastasis-negative (LN−) nodes in different groups.

	Preoperative evaluation	Lower LN group		Upper LN group	Postoperative evaluation
		SLN	Non-SLN		
LN (−)	43	57	53	69	53
LN (+)	29	15	19	3	19
Number of samples	72	72	72	72	72

SLN: sentinel lymph nodes group; non-SLN: non-sentinel lymph nodes group.

**Table 4:** Macro-metastasis location in individual patients.

	Patient ID	Lower LN group		Upper LN
		SLN group	Non-SLN
1*	512961*	−*	+*	−*
2*	534504*	−*	+*	−*
3^#^	540157^#^	−^#^	+^#^	+^#^
4*	595554*	−*	+*	−*
5^$^	515192^$^	+^$^	+^$^	−^$^
6^$^	514209^$^	+^$^	+^$^	−^$^
7^§^	609109^§^	+^§^	+^§^	+^§^
8^$^	587996^$^	+^$^	+^$^	−^$^
9^$^	544091^$^	+^$^	+^$^	−^$^
10^$^	546770^$^	+^$^	+^$^	−^$^
11^$^	533853^$^	+^$^	+^$^	−^$^
12^$^	517425^$^	+^$^	+^$^	−^$^
13^$^	589776^$^	+^$^	+^$^	−^$^
14^$^	533191^$^	+^$^	+^$^	−^$^
15^$^	567965^$^	+^$^	+^$^	−^$^
16^$^	555070^$^	+^$^	+^$^	−^$^
17^§^	584967^§^	+^§^	+^§^	+^§^
18^$^	617172^$^	+^$^	+^$^	−^$^
19^$^	480261^$^	+^$^	+^$^	−^$^

Sum (+)		15	19	3

SLN: sentinel lymph nodes group; non-SLN: non-sentinel lymph nodes group.

**Table 5:** Number of patients with micro-metastasis (macro-metastasis-negative nodes stained with CK-22) in different node groups.

	Pre-operative evaluation	Lower LN Group		Upper LN group	Post-operative evaluation
		SLN	Non-SLN		
LNMM (−)	34	41	29	50	28
LNMM (+)	19	12	24	3	25
Number of samples	53	53	53	53	53

LNMM (−): micro-metastasis negative; LNMM (+): micro-metastasis positive; SLN: sentinel lymph nodes group; non-SLN: non-sentinel lymph nodes group.

**Table 6:** Micro-metastasis (macro-metastasis-negative nodes stained with CK-22) in different node groups for individual patients.

	Patient ID	Lower LN group		Upper LN
		SLN group	Non-SLN group
1^#^	596794^#^	+^#^	+^#^	+^#^
2^§^	524841^§^	+^§^	+^§^	−^§^
3^§^	534928^§^	+^§^	+^§^	−^§^
4^§^	490306^§^	+^§^	+^§^	−^§^
5^§^	580431^§^	+^§^	+^§^	−^§^
6^§^	575327^§^	+^§^	+^§^	−^§^
7^§^	572801^§^	+^§^	+^§^	−^§^
8^§^	511855^§^	+^§^	+^§^	−^§^
9^§^	552392^§^	+^§^	+^§^	−^§^
10^§^	600862^§^	+^§^	+^§^	−^§^
11^§^	606324^§^	+^§^	+^§^	−^§^
12^¥^	566900^¥^	+^¥^	−^¥^	−^¥^
13*	533935*	−*	+*	+*
14*	603455*	−*	+*	+*
15^$^	565404^$^	−^$^	+^$^	−^$^
16^$^	602786^$^	−^$^	+^$^	−^$^
17^$^	481275^$^	−^$^	+^$^	−^$^
18^$^	610052^$^	−^$^	+^$^	−^$^
19^$^	586968^$^	−^$^	+^$^	−^$^
20^$^	581075^$^	−^$^	+^$^	−^$^
21^$^	584976^$^	−^$^	+^$^	−^$^
22^$^	575268^$^	−^$^	+^$^	−^$^
23^$^	473538^$^	−^$^	+^$^	−^$^
24^$^	527037^$^	−^$^	+^$^	−^$^
25^$^	614530^$^	−^$^	+^$^	−^$^

Sum (+)		12	24	3

SLN: sentinel lymph nodes group; non-SLN: non-sentinel lymph nodes group; Sum (+): sum of positive staining nodes.

## Abbreviations

ICBNs: Intercostobrachial nerves
ALND: Axillary lymph node dissection
LNs: Lymph nodes
SLN: Sentinel lymph node
MBD: Methylene blue dye
H&E: Hematoxylin and eosin
CK-22: Cytokeratin-22
ALN: Axillary lymph node
SJHCMU: Shengjing Hospital of China Medical University
IDC: Infiltrating duct carcinoma
AJCC: American Joint Committee of Cancer
MTP: Molybdenum target photograph
PMBTA: Pectoralis minor branch of thoracoacromial artery.
